# Health-related quality of life profile of Indonesian children and its determinants: a community-based study

**DOI:** 10.1186/s12887-022-03161-0

**Published:** 2022-02-22

**Authors:** Mei Neni Sitaresmi, Braghmandita Widya Indraswari, Nisrina Maulida Rozanti, Zena Sabilatuttaqiyya, Abdul Wahab

**Affiliations:** 1 Department of Child Health, Faculty of Medicine, Public Health and Nursing, Universitas Gadjah Mada (UGM)/ RSUP DR Sardjito, Jalan Farmako Sekip Utara, Yogyakarta, 55281 Indonesia; 2 Faculty of Medicine, Public Health and Nursing, Universitas Gadjah Mada (UGM), Yogyakarta, Indonesia; 3 Department of Biostatistics, Epidemiology, and Population Health, Faculty of Medicine, Public Health and Nursing, Universitas Gadjah Mada (UGM), Yogyakarta, Indonesia

**Keywords:** Health-related quality of life, Children, Acute illness, Sociodemographic, Low- and middle-income countries

## Abstract

**Background:**

Assessing health-related quality of life (HRQOL) and its determinants in children may provide a comprehensive view of child health. The study aimed to assess the HRQOL in Indonesian children and its determinants.

**Methods:**

We conducted a community-based cross-sectional study in the Sleman District of Yogyakarta Special Province, Indonesia, from August to November 2019. We recruited children aged 2 to 18 years old using the Sleman Health and Demography Surveillance System sample frame. We used the validated Indonesian version of Pediatric Quality of life Inventory™ (Peds QL™) 4.0 Generic core scale, proxy-reports, and self-reports, to assess the HRQOL.

**Results:**

We recruited 633 proxies and 531 children aged 2–18 years. The mean total score of self-report and proxy-report were 89.9+ 8.5 and 93.3 + 6.4. There was a fair to moderate correlation between self-reports and proxy-reports, with intra-class correlation ranging from 0.34 to 0.47, all *p* < 0.001. Half of the children (49.4% from proxy-report and 50.1% from self-report) reported having acute illness during the last month. Based on proxy-reports, multivariate regression analysis demonstrated lower HRQOL for children with acute health problems, younger age, history of low birth weight, abnormal delivery, lower fathers’ educational level, and government-paid insurance for low-income families.

**Conclusion:**

Sociodemographic determinants of a child’s HRQOL, acute health problems, and low birth weight were associated with lower HRQOL in the general pediatric population. In low- and middle-income countries where acute infections and low birth weight are still prevalent, its prevention and appropriate interventions should improve child health.

## Background

Health-related quality of life (HRQOL) can give a broader aspect of health according to the World Health Organization (WHO) constitution, including aspects of perceived health, psychosocial, mental health, and well-being [1]. Therefore, HRQOL can comprehensively measure child health in the general population and better identify unrecognized conditions, social and emotional problems, and poor functioning [2]. Measuring HRQOL in the pediatric population can help determine the burden of disease and assess the impact of prevention and intervention. Furthermore, it may also identify health disparities, assist in the evaluation of the health care needs of a community, and makes a policy decision for stakeholders [3]. Self-assessed health status is also a more powerful predictor of mortality and morbidity than many objective measures of health [4].

Previous studies showed some determinants of HRQOL in children and adolescents, such as sociodemographic factors, gender, age, and health status. Lower parental income and lower parental educational level were associated with lower HRQOL [5]. The number of health problems and ﻿health care visits are negatively correlated with HRQOL [6, 7]. Perinatal factors such as birth weight and gestation age were related to HRQOL in adolescence [8]. In Indonesia, as a low- and middle-income country, the prevalence of acute illness and low birth weight is still high [9]. LBW has been identified as a risk of adverse outcomes, not only infant mortality-morbidity but also impaired neurodevelopment outcomes at school-age and non-communicable diseases later in life. Acute illness and LBW may be associated with low children’s HRQOL [10]. Therefore, it is crucial to assess the influence of acute disease and low birth weight on HRQOL. The study aimed to evaluate the HRQOL ﻿in children aged 2–18 years in the general population and its determinants.

## Methods

### Study design

We conducted a community-based cross-sectional study in the Sleman District of Yogyakarta Special Province, Indonesia. Sleman District, a semi-urban city, consists of 17 sub-districts and 86 villages and is 574.82 km^2^ wide, with a population of 1,167,481, and 29.5% are under 20 years old [11]. We randomly selected 660 children aged 2–18 years old living in the area of Health and Demographic Surveillance System (HDSS), Sleman. The study aimed to evaluate the HRQOL in children in the general population and focused on the correlation between acute conditions with HRQOL. Therefore, we excluded children with chronic health problems and hospitalized children during data collection. All parents of these children who meet the inclusion and exclusion criteria filled the proxy-report PedsQL questionnaire. However, since the self-report PedsQL is only available for children aged 5–18 years old, we only included those children aged 5–18 years to fill the self-report PedsQL questionnaire. Considering the total population of children and level of confidence 95%, the sample size was 600 subjects. A cluster sampling approach was used to collect the sample from children in The Health and Demographic Surveillance System (HDSS) sample frame in Sleman, Yogyakarta. The Sleman HDSS is a longitudinal household-based survey, and the survey was conducted annually, starting in 2015 [11]. Ethical approval was obtained from the Medical and Health Research Ethics Committee of the Faculty of Medicine, Public Health, and Nursing.

Written informed consent was obtained from all parents/guardians of the children 2–18 years, and assent of 11–18 years old children. Participation was voluntary, and confidentiality was ensured.

### Instruments and measurements

Trained interviewers collected data at a home visit through a Computer-Assisted Personal Interview on a tablet device. The data were transferred to the server through the Internet. The quality control consisted of spot-checks of 5% of interviews to control adherence to the protocol, re-checks to ensure the validity of the interview, and computer-based data cleaning. Sociodemographic data includes parental data on educational level (< 9 years is considered as low education level), working status, living area (urban and rural-based on the listing from the Indonesia statistic), and health insurance ownership (government-paid for low-income families, self-funding for more prosperous families). Children’s data included age, gender, birth weight (birth weight < 2500 g is considered as LBW, mode of delivery, and health condition (if the child suffered from fever, diarrhea, vomiting, cough, and other symptoms during the last month).

HRQOL was assessed using The Indonesian Validated Version of Pediatric Quality of life Inventory™ (PedsQL™) 4.0 Generic Core Scale. A cultural-linguistic validation test has been conducted by Sitaresmi et al. [12]. The PedsQL™ is a multidimensional instrument developed by Varni et al. [13]. It consists of 23 items categorized into four subscales: physical functioning (8 items), emotional functioning (5 items), social functioning (5 items), and school functioning (5 items). For interpretation, three scores can be obtained: physical health (score of the physical functioning subscale), psychosocial health (combined scores of the emotional functioning, social functioning, and school functioning subscales), and the total score (combined score of physical health and psychosocial health). The PedsQL is available for children aged: 5–7, 8–12, and 13–18 years old, as well as for the guardian of children aged: 2–4, 5–7, 8–12, and 13–18 years old. The scale has five Likert response options: never, almost never, sometimes, often, and almost always. To simplify the interpretation, all Likert scales were converted to 0–100. The higher scores indicate a higher HRQOL. There are only three response options for the versions adapted to children between the ages of 5 and 7 years: never, some- times, and almost always (corresponding to 100, 50, 0). For this age, a Face Scale was used, comprising 3 pictures of facial expressions varying from a smiling face to a very sad face to indicate no problem/difficulty to a lot of problems/difficulty [14].

### Statistical analysis

We used EpiData for data management, and data analysis was performed with SPSS version 25 for Windows. Baseline characteristics were first described using mean, median, or proportion as appropriate. Mean presented by calculation of total score of HRQOL for each subject. We compared scores between different demographic characteristics using the Independent-Sample T-Test. To identify variables that most closely predict the HRQOL score, we conducted a backward multiple regression analysis for each PedsQL™ subscale as the dependent variable and sociodemographic characteristic and health status as an independent variable. Analyses were performed with two-sided tests, and a *p*-value < 0.05 was considered significant. Agreement between child self-report and parent proxy-report on the PedsQL™ 4.0 was assessed using Intra-Class Correlation Coefficients (ICC). An ICC lower than 0.4 was considered as very low, 0.4 to 0.74 as low to acceptable, and 0.75 or higher as excellent [15].

## Results

### Participant characteristics

A total of 633 guardians, most of them were mothers (66%), and 531 children were recruited. Table 1 shows that slightly more boys (51.8%) than girls participated, with the mean age being 10.40 ± 4.7 years. Half of the children both from self-report and proxy-report reported suffering from acute illness, with the most common symptoms were cough/coryza (37.8%), fever (12.5%), and diarrhea (2.5%). A higher proportion of 2–12 years old children (56%) suffered from an acute illness compared to the adolescence group (38%). Table 2 shows the mean score of self-reports and proxy-reports. Both from self-reports and proxy-reports, school functioning, was the lowest score. There was a low to moderate agreement between self-reports and proxy-reports, with ICCs ranging from 0.38 to 0.49. (Table 2).

**Table 1:** General Characteristic of Respondents

Variable	Child self-reports	Proxy-reports
	(*n* = 531)	(*n* = 633)
	n (%)	n (%)
**Respondent status**		
Fathers		143 (22.59)
Mothers		420 (66.35)
Other caregivers		70 (11.06)
**Child age in year, mean (SD)**	11.5 (3.9)	10.4 (4.7)
2–12 year-group	300 (56,50)	396 (62.56)
13–18 year-group	231 (43.50)	237 (37.44)
**Child gender**		
Male	281 (52.92)	328 (51.82)
Female	250 (47.08)	305 (48.18)
**Number of children in the family**		
1	82 (15.44)	111 (17.54)
2	266 (50.09)	311 (49.13)
≥ 3	183 (34.46)	211 (33.33)
**Order of the child in the family**		
1st child	231 (43.50)	270 (42.65)
2nd or more	300 (56.50)	363 (57.35)
**Birth weight**		
> 2500 g	480 (90.40)	574 (90.68)
< 2500 g	30 (5.65)	38 (6.00)
Unknown	21 (3.95)	21 (3.32)
**Mode of delivery**		
Normal	455 (85.69)	528 (83.41)
SC	75 (14.12)	104 (16.43)
Unknown	1 (0.19)	1 (0.16)
**History of illness in the past one month**		
Yes	237 (44.63)	313 (49.45)
No	294 (55.37)	320 (50.55)
**Location**		
Urban	458 (86.25)	549 (86.73)
Rural	73 (13.75)	84 (13.27)
**Fathers’ educational level**		
Basic education (< 9 years education)	173 (32.58)	195 (30.81)
Higher education (> 9 years education)	358 (67.42)	438 (69.19)
**Mothers’ educational level**		
Basic education (< 9 years education)	169 (31.83)	195 (30.81)
Higher education (> 9 years education)	362 (68.17)	438 (69.19)
**Working situation parents**		
both parents work	245 (46.14)	285 (45.02)
one parent work or both parents do not work	286 (53.86)	348 (54.98)
**Insurance**		
Paid by government or do not have Insurance	317 (59.69)	371 (58.61)
self- funded	214 (40.31)	262 (41.39)

**Table 2:** Intra-Class Correlation between self-report and proxy-report

Scale descriptive	Child self-reports	Proxy-reports	ICC	*p*
	Mean (SD)	Mean (SD)		
**Total sample (n)**	531	633		
Total score	89.28 (8.92)	92.90 (6.73)	0.49	< 0.000
Physical health	94.24 (7.65)	96.35 (6.27)	0.45	< 0.000
Psychosocial health	87.63 (10.30)	91.71 (8.02)	0.48	< 0.000
Emotional functioning	85.19 (15.39)	89.24 (12.07)	0.41	< 0.000
Social functioning	93.14 (10.873)	96.49 (7.65)	0.38	< 0.000
School functioning	84.39 (13.80)	89.35 (11.56)	0.48	< 0.000

*SD* Standard deviation

### Bivariate analysis

Table 3 shows that the number of children, child position in the family, father educational level, working parents’ status, health insurance ownership, and child health status are significantly related to HRQOL based on the self-report. Table 4 shows that child age, birth weight, mode of delivery, father education level, health insurance ownership, and child health status are significantly related to HRQOL based on the proxy report. A higher total mean score was found in children without a history of acute illness during the past last month, history of normal birth weight, normal delivery, higher level of father’s education, and self-funding Insurance based on the proxy report. Both children and their guardians did not report any significant gender differences in HRQOL.

**Table 3:** Bivariate analysis of health-related quality of life’s determinants based on child report

Scale Description	Total score	Physical health	Psychosocial health	Emotional functioning	Social Functioning	School Functioning
	Mean (SD)	Mean (SD)	Mean (SD)	Mean (SD)	Mean (SD)	Mean (SD)
**Age Group**						
Children group (2–12 Years) (*n* = 300)	88.95 (9.08)	94.29 (7.67)	87.19 (10.62)	84.20 (15.85)**	92.38 (11.69)*	85.00 (14.54)
Adolescence group (13–18 Years) (*n* = 231)	89.71 (8.70)	94.17 (7.65)	88.21 (9.87)	86.47 (14.71)	94.11 (9.64)	83.55 (12.71)
**Child gender**						
Male (*n* = 281)	89.39 (8.71)	94.55 (6.35)	87.65 (10.40)	86.10 (15.33)	93.10 (10.78)	83.46 (13.71)
Female (*n* = 250)	89.16 (9.16)	93.89 (8.88)	87.60 (10.21)	84.16 (15.43)	93.18 (11.00)	85.41 (13.85)
**Number of children in the family**						
2 (*n* = 348)	89.87 (8.17)*	94.63 (7.04)	88.29 (9.54)*	86.05 (14.33)	93.38 (9.87)	85.31 (13.05)*
> 3 (183)	88.17 (10.12)	93.49 (8.65)	86.38 (11.53)	83.55 (17.15)	92.68 (12.58)	82.61 (15.02)
**Position of the child in the family**						
1st child (*n* = 231)	90.31 (7.48)*	94.80 (6.56)	88.82 (8.71)*	86.13 (14.36)	94.33 (8.72)*	85.84 (12.22)*
2nd or more (*n* = 300)	88.12 (10.00)	93.67 (8.29)	86.27 (11.58)	83.89 (16.59)	91.55 (13.00)	83.38 (14.68)
**Birth weight**						
< 2500 g (*n* = 30)	86.73 (10.05)	92.08 (9.38)	84.94 (10.84)	82.17 (15.69)	91.33 (12.38)	80.86 (12.68)
> 2500 g (*n* = 480)	89.34 (8.89)	94.29 (7.64)	87.69 (10.30)	85.20 (15.45)	93.20 (10.83)	84.51 (13.90)
**Mode of delivery**						
Normal (*n* = 455)	89.44 (8.90)	94.48 (7.49)	87.76 (10.33)	85.27 (15.55)	93.18 (10.97)	84.65 (13.83)
SC (*n* = 75)	88.21 (9.02)	92.71 (8.48)	86.71 (10.18)	84.47 (14.49)	92.80 (10.41)	82.67 (13.64)
**History of illness in the past one month**						
Yes (*n* = 237)	87.50 (9.22)**	92.76 (8.09)**	85.75 (10.75)**	83.48 (16.23)*	92.09 (11.37)*	81.59 (14.24)**
No (*n* = 294)	90.72 (8.41)	95.43 (7.06)	89.15 (9.68)	86.56 (14.56)	93.98 (10.40)	86.65 (13.02)
**Location**						
Urban (*n* = 458)	89.02 (8.99)	94.10 (7.72)	87.32 (10.37)	84.88 (15.36)	92.97 (10.84)	84.04 (14.09)
Rural (*n* = 73)	90.95 (8.31)	95.08 (7.22)	89.58 (9.72)	87.12 (15.52)	94.18 (11.09)	86.69 (11.51)
**Fathers’ educational level**						
Basic education (*n* = 173)	88.19 (9.81)	93.42 (8.78)	86.45 (11.19)	85.61 (15.25)	91.47 (12.85)*	81.79 (14.35)**
Higher education (*n* = 358)	89.81 (8.41)	94.63 (7.02)	88.20 (9.81)	84.99 (15.47)	93.94 (9.69)	85.61 (13.38)
**Mothers’ educational level**						
Basic education (*n* = 169)	89.17 (9.05)	94.12 (8.28)	87.56 (10.24)	86.33 (14.43)	92.04 (12.08)	83.62 (14.34)
Higher education (*n* = 362)	89.33 (8.87)	94.29 (7.35)	87.66 (10.34)	84.65 (15.81)	93.65 (10.24)	84.73 (13.56)
**Working parents**						
Both (*n* = 245)	90.08 (8.47)	95.00 (6.90)*	88.43 (9.82)	86.29 (14.99)	93.57 (10.25)	85.27 (12.84)
One or none (*n* = 286)	88.60 (9.24)	93.59 (8.20)	86.95 (10.67)	84.25 (15.69)	92.76 (11.38)	83.63 (14.56)
**Insurance**						
Paid by government or have no insurance (*n* = 317)	88.78 (9.36)	93.57 (7.96)*	87.17 (10.83)	84.53 (16.60)	92.95 (10.84)	83.88 (14.07)
Self-funding (*n* = 214)	90.03 (8.17)	95.22 (7.07)	88.32 (9.45)	86.17 (13.39)	93.41 (10.94)	85.14 (13.40)

*p < 0.05 **p < 0.01; SD, standard deviation

Analysis using Independent -sample T-test

**Table 4:** Bivariate analysis of health-related quality of life’s determinants based on proxy-report

Variable	Total score	Physical Functioning	Psychosocial Functioning	Emotional Functioning	Social Functioning	School Functioning
	Mean (SD)	Mean (SD)	Mean (SD)	Mean (SD)	Mean (SD)	Mean (SD)
**Age**						
Children (2–12 Years) (*n* = 396)	91.77 (7.42)**	96.05 (6.84)	90.29 (8.89)**	87.50 (12.90)**	95.04 (9.03)**	88.34 (12.68)*
Adolescence (13–18 Years) (*n* = 237)	94.78 (4.82)	96.86 (5.16)	94.10 (5.58)	92.15 (9.90)	98.90 (3.30)	90.97 (9.28)
**Child gender**						
Male (*n* = 328)	92.86 (6.57)	96.78 (5.00)	91.50 (7.88)	89.18 (12.27)	96.89 (7.12)	88.29 (12.18)*
Female (*n* = 305)	92.94 (6.90)	95.89 (7.38)	91.94 (8.18)	89.31 (11.88)	96.05 (8.16)	90.46 (10.78)
**Number of children in the family**						
< =2 (*n* = 422)	92.76 (7.11)	96.25 (6.70)	91.56 (8.44)	89.18 (12.54)	96.40 (7.59)	89.15 (11.90)
> 2 (*n* = 211)	93.17 (5.90)	96.55 (5.31)	92.02 (7.12)	89.36 (11.10)	96.66 (7.77)	89.75 (10.86)
**Child position in the family**						
1st child (*n* = 270)	92.92 (6.97)	96.28 (6.14)	91.77 (8.22)	89.07 (12.80)	96.70 (7.43)	89.60 (10.88)
2nd or more (*n* = 363)	92.88 (6.55)	96.40 (6.38)	91.67 (7.88)	89.37 (11.52)	96.32 (7.81)	89.15 (12.08)
**Birth weight**						
< 2500 g (*n* = 38)	90.49 (8.55)*	94.74 (6.70)	89.09 (10.44)*	83.82 (17.02)**	95.00 (9.72)	87.96 (11.94)
> 2500 g (*n* = 574)	93.02 (6.56)	96.39 (6.32)	91.86 (7.77)	89.53 (11.65)	96.59 (7.43)	89.44 (11.45)
**Mode of delivery**						
Normal (*n* = 528)	93.20 (6.58)**	96.49 (6.48)	92.10 (7.70)**	89.74 (11.50)*	96.76 (7.39)*	89.61 (11.37)
SC (*n* = 104)	91.33 (7.25)	95.64 (5.10)	89.72 (9.29)	86.68 (14.51)	95.05 (8.77)	87.73 (12.54)
**History of illness in the past one1 month**						
Yes (*n* = 313)	91.02 (7.43)**	94.95 (7.34)**	89.67 (8.72)**	86.44 (13.27)**	95.42 (8.55)**	87.01 (12.59)**
No (*n* = 320)	94.73 (5.36)	97.72 (4.63)	93.71 (6.71)	91.98 (10.07)	97.53 (6.49)	91.57 (10.02)
**Location**						
Urban (*n* = 549)	92.84 (6.81)	96.37 (6.39)	91.63 (8.10)	89.14 (11.97)	96.47 (7.82)	89.29 (11.67)
Rural (*n* = 84)	93.28 (6.19)	96.21 (5.43)	92.24 (7.51)	89.88 (12.82)	96.61 (6.41)	89.80 (10.88)
**Fathers’ educational level**						
Basic education (*n* = 195)	91.83 (7.31)**	95.66 (7.40)	90.55 (8.78)*	87.90 (13.36)	96.08 (8.74)	87.35 (12.04)**
Higher education (*n* = 438)	93.37 (6.40)	96.66 (5.67)	92.23 (7.61)	89.84 (11.42)	96.67 (7.11)	90.24 (11.25)
**Mothers educational level**						
Basic education (*n* = 195)	93.47 (6.05)	96.27 (7.44)	92.56 (7.08)	90.31 (11.03)	97.15 (6.73)	89.65 (10.99)
Higher education (n = 438)	92.64 (7.00)	96.39 (5.68)	91.33 (8.39)	88.77 (12.50)	96.19 (8.01)	89.22 (11.81)
**Working parents**						
Both (*n* = 285)	93.27 (6.63)	96.36 (5.34)	92.19 (8.03)	89.40 (12.42)	96.89 (7.61)	90.39 (11.10)*
One or none (*n* = 348)	92.59 (6.80)	96.35 (6.95)	91.32 (8.00)	89.11 (11.80)	96.15 (7.68)	88.49 (11.88)
**Insurance**						
Paid by government or have no insurance (*n* = 371)	92.42 (6.84)*	96.04 (6.06)	91.16 (8.07)*	88.91 (11.98)	96.15 (7.95)	88.37 (11.37)*
Self- funding (*n* = 262)	93.57 (6.52)	96.80 (6.54)	92.49 (7.90)	89.71 (12.21)	96.97 (7.18)	90.69 (11.22)

*p < 0.05 **p < 0.01

Analysis using Independent -sample T-test, *SD* Standard deviation

### Multivariate analysis

In linear regression models, variables included in the final model for the total mean score of child-report were child health status, number of children, living area, and father education level. Children who suffered from acute illness during the last month, children with lower fathers’ education level, and who live in an urban area perceived lower HRQOL than their counterparts. The final regression model for the total mean score for proxy-reports included children’s age, health status, birth weight, mode of delivery, fathers’ education level, and insurance ownership. The coefficients and corresponding confidence intervals are presented in Table 5.

**Table 5:** Multivariate analysis of HRQOL Total score: self-report and proxy report

Variable	Self-report (total score) ***N*** = 510		Proxy report (total score) ***N*** = 612	
	Coefficient	CI 95%	Coefficient	CI 95%
Age (2 to 12 years old = Ref)			2.55**	1.49 to 3.61
Number of children (2 = Ref)	−1.78*	−3.28 to −0.17		
History of illness in the past 1 month (healthy = Ref)	−2.98**	−4.52 to − 1.45	−3.00**	−4.02 to − 1.98
Birth weight (> 2.500 g = Ref)			− 2.46*	− 4.54 to − 0.37
Mode of delivery (vaginal delivery = Ref)			−1.45*	−2.80 to − 0.09
Living area (urban = ref)	2.91*	0.69 to 5.10		
Fathers’ educational level (higher level = Ref)	−1.73*	−3.38 to − 0.08	− 1.66*	−2.75 to − 0.56
Insurance (self-funding = Ref)			− 1.20*	− 2.22 to − 0.19
	adjusted R^2^ .060		adjusted R^2^ .128	

* *p* < 0.05; **p < 0.01

*CI* Confidence interval

## Discussion

Measuring HRQOL and its determinants in the pediatric population can provide insight into developing more targeted public health policies planned by multiple stakeholders [16]. To our knowledge, this study was the first study assessing HRQOL and its determinants in the general pediatric population in Indonesia. Surprisingly, the total score of HRQOL in our general pediatric population is higher than those in the references study by Varni et al., 89 + 9 vs. 84 + 12 (self-reports) and 92 + 6 vs. 82 + 15 (proxy-reports) [3]. However, the mean total score of our study was similar to the scores from healthy children in India (87 ± 11) for self-reports and 90 ± 9 for proxy-reports [17]. According to WHO, quality of life is a personal perception of their position in life in the context of their culture and value system and related to their goals, expectations, values, and concerns [18]. The high score of HRQOL in our study may be explained by the fact that our population positively perceived and was more satisfied with their lives. A study by Jaafar et al. showed that Indonesian adolescents had significantly higher happiness, gratitude, and resilience levels than neighbouring countries [19]. According to a survey conducted by a UK-based charity foundation, Indonesian youths topped the list among the happiest in 20 countries. In addition, the survey reported that Indonesian youths had better emotional wellbeing, less anxiety, and strong religious faith [20, 21].

In both self-reports and proxy-reports, the school functioning was the lowest score among the other domains. It may be explained that almost half of our respondents, especially in the age of 2–12 years, had an acute illness. It may result in school absences and lower the school score. This finding is similar to the study assessing HRQOL of children with acute febrile illness [16] and in bronchitis children [22], which used the PedsQL™ and found the school functioning was the lowest score.

### Sociodemographic determinant of child HRQOL

We found some sociodemographic characteristics related to children’s HRQOL. Children who were living in the rural area reported better HRQOL than their urban counterparts. This is remarkable, considering the rural area is associated with a low-resource setting. The finding may probably be due to different perceptions or expectations about HRQOL and life amenities between rural and urban children [17]. As reported by a previous study [23], we found that children with a higher father’s education level had higher HRQOL, both from the self-reports and proxy-reports. Higher levels of parents’ education might increase parents’ awareness and knowledge about their children’s health and support getting better occupations and income, leading to better HRQOL and life expectancy [23].

On the contrary, we did not find any influence of maternal educational level on children’s HRQOL. It may be explained by the fact that fathers still hold the leading role in most of Indonesia’s families. Indonesia is using a patriarchal system which is still common in society and generally accepted by the community. This system raises the principle and value that all the family’s decisions, including children’s education and health-seeking behavior, are managed by the fathers [24].

A previous study found the health insurance ownership influenced a child’s HRQOL [25]. We found that children from self-funding insurance families had higher HRQOL scores than children from families with government-paid insurance or no insurance, both from proxy-reports as well as self-reports. In our population, health insurance ownership may indirectly reflect family socioeconomic status. Since 2014, the Indonesian government has introduced National Health Insurance. This obligatory national health insurance system combines government-paid health insurance for low-income families and contributory-based (self-funding) health insurance for the more prosperous families [26]. In addition, people with health insurance receive more appropriate and recommended use of health services and have better health outcomes. In contrast, people with no insurance are less likely to receive preventive and screening services, regular and continuing sources of care [27].

Previous study that found declining HRQOL with increasing age [28]. Interestingly, our adolescent group had higher HRQOL than younger children. However, the higher HRQOL in the adolescent group was only in the proxy-report group, which included children aged 2–4 years. In the self-report group, which consists of children older than 5 years, there was no significant difference in HRQOL between the younger and adolescent groups. It may be related to the higher prevalence of acute disease in younger Indonesia’s children, especially under-five [9]. We found a higher proportion of 2–12 years old children (56%) suffered from an acute illness than the adolescent group (38%).

### Child health determinants on child HRQOL

Most studies assess chronic health problems’ influences on a child’s HRQOL [6, 16, 23], but few studies assessed the influence of acute illness. A study on determinants of HRQOL in Dutch school-age children’s in the general population found that children who had acute health complaints showed lower scores of HRQOL [6]. Our study found that the best predictor of children’s HRQOL was the presence of acute illness during the past month. In multivariate regression analysis, we consistently found that having an acute disease, such as upper respiratory symptoms and diarrhea, during the past month was associated with a lower score of the child’s HRQOL from the self-reports and proxy-reports across all domains. During the past month, more than one-third of our children have cough or/and coryza, with or without fever. We excluded children who were hospitalized during the past last month. Our sample represented that upper respiratory tract symptoms are a prevalent child health problem in our setting. A most acute cough is due to an upper respiratory tract infection (URTI) [29]. Even though URTI is associated with low mortality, it significantly disturbs children’s daily activities such as sleeping and eating and may cause school absences and parent work absences [30]. A previous study assessing the effect of acute cough on child HRQOL using the Parent-proxy Children’s Acute Cough-specific QoL (PAC-QoL) Questionnaire found that children who suffered acute cough have lower HRQOL across all domains [31].

We found that perinatal factors, namely LBW and abnormal delivery, were associated with a lower the child’s HRQOL based on proxy-reports. This result was similar to a review study assessing the impact of preterm and LBW on HRQOL of preschool- and school-aged children, adolescents, and young adults. The review found that the history of prematurity and LBW lower the HRQOL at various age groups. The effect of LBW and gestational age is greatest during the younger period, but the effect extended into adulthood [32]. Our finding is quite essential since LBW is still a significant public health problem. In 2015, an estimated 20.5 million live births were LBW, 91% from low-middle income countries, mainly southern Asia (48%) and sub-Saharan Africa (24%) [33]. The prevalence of LBW in our study population was 5.6% (self-reported) and 6.0% (proxy-reported), similar to the prevalence of LBW reported by Indonesian Basic Health Research 2018, which was 6.2% [9]. LBW has been identified as a risk of adverse outcomes other than infant mortality-morbidity, including impaired neurodevelopment outcome at school-age and non-communicable diseases later in life [10]. A systematic review and meta-analysis studies on the impact of LBW on South Asian children found that children born with LBW have significantly impaired cognitive and motor function [34]. Our study provides an additional value to use HRQOL assessment, a simple but valid and reliable tool, to detect the impact of perinatal problems on the whole aspect of health in the preschool to adolescence period. Our finding also emphasized the importance of preventing LBW.

### Strengths and limitations

This study has some limitations. This study was a cross-sectional study; therefore, the results only support an association between determinant variables and HRQOL, not causality. In addition, other determinants like school environment were not assessed. Thus further research is needed. A study in urban schoolchildren found that children’s perception of closeness to school personnel and the school environment and “school connectedness’ were significantly related to the HRQOL [25]**.** We excluded children who have been diagnosed with chronic health problems, mental and behavioral health problems. However, a cluster sampling approach using the Health and Demographic Surveillance System (HDSS) sample frame makes this study represent HRQOL of “healthy” children in the semi-urban city of Indonesia. Including acute health complaints and LBW, common health problems in developing countries, as determinants of children’s HRQOL, support the importance of health promotion and prevention. Using both proxy-reports and self-reports is also the strength of the study. We found there was a fair to moderate agreement between self-reports and proxy-reports. Self-report is considered the standard for measuring HRQOL because it is more likely to represent internal health measures than proxy reporting accurately [35, 36]. However, parent proxy-report should be considered as a secondary measure contributed to health-seeking behavior or health care usage [37].

## Conclusions

Sociodemographic determinants of a child’s HRQOL, acute health problems, and LBW were associated with lower HRQOL in the general pediatric population. In low- and middle-income countries where acute infections and LBW are still prevalent, its prevention and appropriate interventions should improve child health.

## Data Availability

The datasets that support the conclusions of this article are available by request to the corresponding author. We do not make participants’ data publicly available due to data protection restrictions and participant confidentiality.

## Abbreviations

HDSS: The Health and Demographic Surveillance System
HRQOL: health-related quality of life
ICC: Intra-Class Correlation
LBW: Low birth weight
PAC-QoL: Parent-proxy Children’s Acute Cough-specific QoL
Peds QL™: Pediatric Quality of life Inventory™
SPSS: Statistical Package for the Social Sciences
URTI: Upper respiratory tract infection
WHO: World Health Organization
